# The Effect of PEI-Mediated *E1A* on the Radiosensitivity of Hepatic Carcinoma Cells

**DOI:** 10.31557/APJCP.2020.21.4.911

**Published:** 2020-04

**Authors:** Danghui Xu, Jianxin Yao, Yiwen Zhang, Nan Xiao, Peng Peng, Zhanfeng Li, Zhiyao Pan, Zhifeng Yao

**Affiliations:** 1 *Department of Radiology, Affiliated Hospital of Nanjing University of Chinese Medicine, Jiangsu Provincial Hospital of Traditional Chinese Medicine, *; 2 *Department of Medical Imaging, Nanjing Vocational Health College, *; 3 *Department of Nursing, The Affiliated Children's Hospital of Nanjing Medical University, *; 4 *Department of Nursing, Nanjing Health College of Jiangsu Union Technical Institute, *; 6 *Department of Oncology, Nanjing First Hospital, Nanjing Medical University, *; 7 *Department of Radiotherapy, The Second Affiliated Hospital of Nanjing Medical University, Nanjing, Jiangsu Province, *; 5 *Department of Basic Medical Science, Zhejiang University Medical College, Hangzhou, Zhejiang Province, China. *

**Keywords:** Human hepatic carcinoma cell, adenovirus 5 early region 1A, polyethyleneimine, radiosensitivity

## Abstract

**Objective:**

The study was undertaken to investigate the effects of polyethyleneimine (PEI)-mediated adenovirus 5 early region 1A (*E1A*) on radiosensitivity of human hepatic carcinoma cell in vitro and to disclosure the underlying mechanism.

**Materials and Methods:**

Human hepatic carcinoma SMMC-7721 cell line was transfected with *E1A* gene using PEI vector. Untransfected cells (SMMC-7721 group), cells transfected with blank-vector (SMMC-7721-vect group), and cells transfected with E1A gene (SMMC-7721-E1A group) were treated with 6 MV X-ray irradiation at doses of 0, 1, 2, 4, 8 and Gy, respectively. Radiosensitivity was determined by MTT assay and quantified by calculating the cell survival rate. Cell-cycle distribution and apotosis rate were monitored by flow cytometry.

**Results:**

The survival rate of SMMC-7721-E1A was significantly lower than that of SMMC-7721 cell. Apoptosis rate of SMMC-7721-E1A group was significantly higher than that of SMMC-7721group (P<0.01).The ratio of S stage in cell cycle of SMMC-7721-E1A was significantly lower than that in SMMC-7721 cell. The ratio of G2/M stage in cell cycle of SMMC-7721-E1A was significantly higher than that in SMMC-7721 cell (P<0.01).

**Conclusion:**

PEI could transfect* E1A* gene into hepatic carcinoma cells PEI-mediated *E1A* could effectively enhance radiosensitivity of hepatic carcinoma cells which may be related to its effects on apoptosis promoting leading to S phase suppression and G2/M phase arrest**.**

## Introduction

Although great progress has been made in cancer therapy in the past years, liver cancer still remains one of the deadliest malignancies. Hepatocellular carcinoma (HCC) is the most frequent type of human liver cancer and the ﬁfth most common neoplasm worldwide. It represents the third leading cause of cancer-related death and has a high propensity toward recurrence and metastasis (Yang et al., 2018). According to the Cancer Statistics Review of the Surveillance, Epidemiology, and End Results (SEER), the 5-year relative survival rate of cancer originating from the liver and bile duct was only 14.0% from 1999 to 2005 in the United States (Jemal et al., 2010). Therefore, it seems that new therapeutic methods with high efficiency and safety are urgently demanded. 

Gene therapy have shown promising efficacy in the treatment of various malignancies. With the development of vector systems, tumor-specific oncolytic adenovirus, which replicates selectively in tumor cells, has become an appealing treatment strategy (Zhang et al., 2017). Thus, gene therapy can be one of the most promising and extensively studied methods in cancer therapy. Adenovirus 5 early region 1A (*E1A*) is the first viral gene expressed in a cell after adenovirus infection. Proteins encoded by *E1A* activate viral transcription and reprogramme cellular gene expression, creating an optimal environment for viral replication (Flint and Shenk, 1989). The primary functions of *E1A* were to induce the cells to enter S phase, deregulate cellular gene expression to favor viral replication, and activate the expression of viral transcription units (Frost et al., 2018).

E1A has oncogenic properties in rodent cells, which can effectively immortalize rodent cells or transform them in cooperation with other oncogenes, such as adenovirus *E1B* gene (Gallimore and Turnellm, 2001). Despite extensive studies ; however, there has been no evidence showing that *E1A* is associated with human malignancies. In previously transformed human and animal cells, *E1A* has demonstrated its ability as an anti-oncogene. It is able to trigger apoptosis, suppress oncogenesis, angiogenesis, and cancer metastasis in vivo, and induce cell differentiation to an epithelium-like type (Mymryk, 1996; Deng et al., 2002; Frisch and Mymryk, 2002). Besides its tumor suppressor activities, *E1A* expression in stably transfected human cancer cell lines has also been reported to increase sensitivity to several apoptotic stimuli, including ionizing radiation, different classes of chemotherapeutic drugs, and tumor necrosis factor –α (TNF-α) (Liao et al.,2007).

The mostly studied vector of gene delivery for* E1A *is adenovirus. Oncolytic adenoviral expressing *E1A*, as monotherapy, is effective against tumor cell lines and xenograft tumor models (Yu et al., 1999; Hoti et al., 2007; Itamochi et al., 2007; Rojas et al., 2010), but only marginal benefit has been seen to date in clinical trials (Chiocca et al.,2004; Small et al.,2006). Gene therapy, as a single agent, is unlikely to succeed in improving overall treatment outcome (Hingorani et al., 2007). Combining gene therapy with other methods such as radiation and chemotherapy may be a better option, which can increase therapeutic efficacy and potentially reduce side effects.

Several preclinical trials have reported that oncolytic adenoviral gene therapy can enhance the therapeutic effect of radiation in treating human prostate cancer, lung adenocarcinoma, thyroid carcinoma, and glioblastoma (Portella et al., 2003; Toth et al., 2003; Dilley et al., 2005; Idema et al., 2007). CV706 and CV787 are both conditionally replicating adenovirus with *E1A* expression. In prostate tumor model, combining CV-706 with radiation has been associated with better tumor control, increased necrosis and apoptosis, and decreased tumor vascularization (Chen et al., 2001). The combination of CV787 with radiation has exhibited synergism anti-tumor efficacy both in vitro and in vivo (Dilley et al., 2005).

Although adenovirus vectors are widely used and are efficient in the delivery of *E1A* into tumor cells in vitro and in vivo, the critical safety issues call for research in nonviral alternatives. Polyethyleneimine (PEI) is considered as a promising candidate for its good biologic performance, low cost, available in large scale, and easy to develop functionalization (Kleemann et al., 2005; Banerjee et al., 2006).

In the present study, the results demonstrated that PEI led to effective delivery and subsequent expression of *E1A* in human hepatoma cell line SMMC-7721, as well as inducing apoptosis. Enhanced radiosensitivity was also shown in *PEI-E1A *gene therapy when combined with ionizing radiation in vitro.

## Materials and Methods

*Cell Culture and Reagents*

Human hepatoma cell line SMMC-7721 was obtained from Liver Cancer Institute of Zhongshan Hospital (Shanghai, China). Cells were cultured in RPMI 1640 (Gibco, USA), supplemented with 10% fetal bovine serum (Gibco) and 1% penicillin-streptomycin (Gibco) at 37°C, and incubated in a humidified atmosphere containing 5% CO_2_.

*Plasmid DNA (pDNA)*

E1A cDNA fragment covering entire coding region (380bp) was excised with Xho and Hind from PsuCMV-E1A plasmid from Dr. CQ Su (Orient Hospital, Shanghai, China) and inserted into mammalian expression vector plasmid pcDNA3.1 with an ampicillin selectable gene. The plasmid pcDNA3.1 without the E1A gene was used as an empty plasmid.

*PEI-pDNA Formulations*

PEI (25 kDa, branched form) was obtained from Shanghai Institute of Applied Physics (Chinese Academy of Sciences, Shanghai, China). The desired amount of pcDNA3.1 in PBS was commixed with PEI by slowly adding the pcDNA3.1 to the PEI while vigorously stirring the solution. The solution was then incubated at room temperature for 30 minutes before use. The resulting charge ratio was expressed as PEI nitrogen: pcDNA3.1 phosphorous (N: P). PEI- pcDNA3.1 was used at a 10:1 N: P ratio, a 5:2 PEI: DNA weight ratio.

*Preparation of PEI-pcDNA3.1-E1A Complexes*

First, 8μl of 10 μg/μl pcDNA3.1-E1A in water was mixed with the equal volume of PEI varying in their concentrations and incubated for 30 min at room temperature. The mixing ratio of PEI and pcDNA3.1-E1A was expressed at a 10:1 N: P ratio, a 5:2 PEI: DNA weight ratio.

*In Vitro Transfection*

For serum-free transfections, cells were plated at 5×10^5^ cells /well in 6-well plates the day before transfection. Cells were approximately 60-70% confluent at the time of transfection. The PEI-DNA formulations were brought up in RPMI1640 supplemented with 10% FBS as above to a final DNA concentration of 1μg/ml. Cells were overlaid with 1 ml of the transfection solution per well. After 6 hours, the transfection solution was removed and the cells were rinsed two times with PBS and overlaid with RPMI1640 supplemented with 10% FBS.

*X-Ray Irradiation Treatment*

Cells were grown and maintained in suitable media, and those reaching 80% confluent were irradiated in media with different doses of gamma radiation ranging from 1-8 Gy from a linear accelerator X-Ray source (Second Affiliated Hospital of Nanjing Medical University, China).

*Flow cytometry*

To quantitatively assess induced apoptotic cell death rate, Annexin V-FITC apoptosis detection assay was performed according to the protocol presented by the manufacturer (Bipec Biopharma Corporation, USA). Briefly, 24 h and 72 h after irradiation, cells were harvested and then resuspended in 400 μL with 1 × binding buffer at a concentration of 1 × 10^6^ cells/ml prior to addition of 5 µL of Annexin V–FITC. Cells were then gently vortexed and incubated for 15 min at 4–8°C in the dark. Next, 10 µL of propidium iodide (PI) was added to each tube before incubation for another 5 min at 4–8°C in the dark. The stained cells were analyzed using a flow cytometer and labeled as viable (annexin V and PI negative), early apoptotic (annexin V positive and PI negative), or late apoptotic (annexin V and PI positive). Use of Annexin-V in combination with PI allowed the distinction of early apoptotic and necrotic cells from viable cells (Yao et al., 2014). The degree of apoptosis was quantified as a percentage of annexin V-positive cells.

For cell cycle analysis, cells were collected and fixed with 70% ethanol in PBS. Propidium iodide was added (20μg/mL), and samples were treated with RNase (20 U/mL). Samples were analyzed by EPICS XL flow cytometer (Coulter Electronics, Hialeah, FL).

*Methyl tetrazolium (MTT) bromide mitochondrial activity assay*

Cell viability was measured by the methyl tetrazolium (MTT) bromide mitochondrial activity assay as described previously (de Loosdrecht et al., 1994). Briefly, 40,000- 50,000 cells/well in 100 µL of medium were seeded in a 96-well plate for 24 h prior to gamma radiation. After 24 h, 10 µL of 5 mg/ mL MTT reagent was added to each well and incubated for 4 h. After incubation, 100 µL of detergent reagent was added to each well to dissolve formazan crystals. The absorbance was determined at 490 nm. Each assay was performed in triplicate, and then the standard deviation was determined.

*Reverse transcription polymerase chain reaction (RT-PCR)*

RNA was isolated from the cultured cells using TRIzol (Invitrogene, USA) following the manufacturer’s protocol. Single-stranded cDNA was prepared with Superscript First-Strand System (Invitrogen) as described previously (Hong et al., 2000). Specific oligonucleotide primers were the forward primer 5’-cggaggtgttattaccgaag-3’ and backward primer 5’-tcgtcactgggtggaaagcc-3’. The PCR reaction consisted of 94 °C for 4 min, 35 cycles at 94°C for 15 s, 60°C for 60 s, and 72°C for 1 min, followed by an extension of 5 min at 72°C. The PCR products were separated by electrophoresis in 1% agarose gels. The length of the expected product was 870bp.

*Western Blot Analysis*

Cells were washed thrice with PBS and then lysed in lysis buffer. Protein content was determined against a standardized control by using a Bio-Rad protein assay kit (Bio-Rad Laboratories, USA). A total of 50 μg of protein was separated by 10% SDS-PAGE and transferred to a polyvinylidene difluoride membrane (Bio-Rad). Nonspecific binding on the membrane filter paper was minimized by blocking buffer consisting of 5% nonfat dry milk and 0.1% (v/v) Tween 20 in PBS. The treated filter paper was then incubated, first with the primary antibody and then with the secondary antibody (horseradish peroxidase–conjugated goat anti-mouse antibody; 1:5000 dilution; Jackson ImmunoResearch Laboratories, USA). The specific used antibodies were mouse anti-E1A antibody (1:500 dilution; PharMingen, USA) and rabbit antiactin antibody (1:5000 dilution; Sigma, USA).

*Statistical analysis*

All data were analyzed by using SPSS (version 16.0) and expressed as mean ± SD. The statistical significance of differences in cell cycle phases and cell viability between SMMC-7721, SMMC-7721-vector, and SMMC-7721-E1A cells was calculated with the Student’s t-test. P<0.05 was considered statistically significant.

## Results

*Transfection of SMMC-7721 cells in vitro by PEI-pcDNA3.1-E1A complexes*

**Figure 1:**
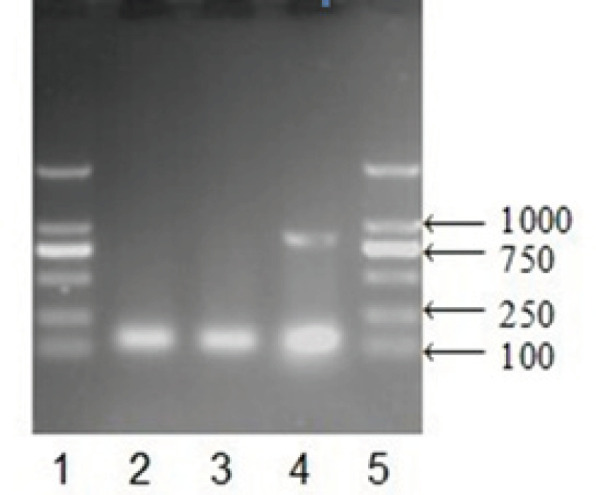
RT-PCR Analysis for E1A Gene (870 bp) Expression in E1A Transfected Cells. Notes: 1, 5: Mark; 2: SMMC-7721 group; 3: SMMC-7721-vect group; 4: SMMC-7721-E1A group

**Figure 2:**
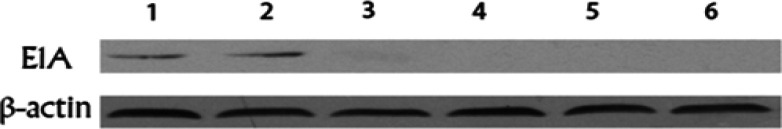
Western Blot Analysis for E1A Protein Expression in SMMC-7721 Cells, β-actin Expression was Shown as a Loading Control. Notes: 1, 2: SMMC-7721-E1A group; 3, 4: SMMC-7721-vect group; 5, 6: SMMC-7721 group

*E1A* expression was determined by RT-PCR and western blot analysis to ensure that the transfection was successful and complete before using *E1A* transfected cells for further analysis. RT-PCR showed detection of 870 bp *E1A* gene expression in *E1A* transfected cells (Figure 1). As shown in Figure 2, E1A transfected cells successfully expressed *E1A*, while empty control plasmid cultured cells or blank control cells did not show any band correspondings to E1A protein.

*Combination of E1A with radiation and its effect on cytotoxicity*

**Figure 3:**
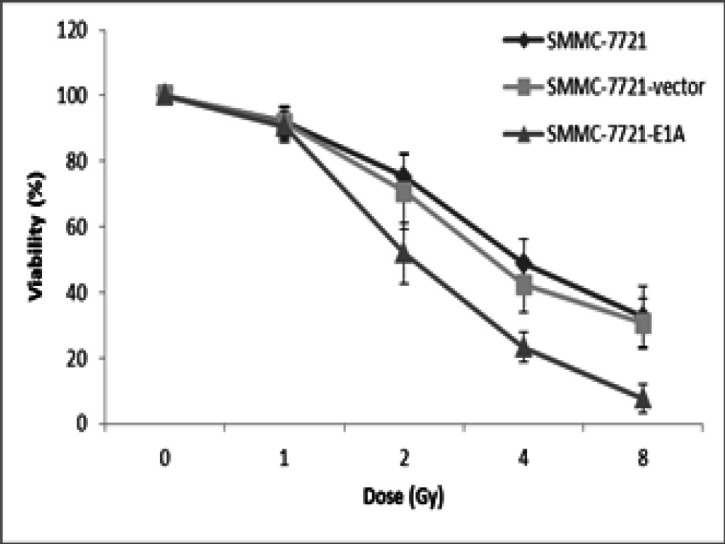
SMMC-7721, SMMC-7721-Vector and SMMC-7721-E1A Cells were Irradiated with Different Doses of Gamma Radiation Ranging from 1-8 Gy. After 72h irradiation, cell viability was determined by MTT assay

To conﬁrm the radiosensitizing effect of PEI-pcDNA3.1-E1A, SMMC-7721 cells transfected with plasmid-E1A (SMMC-7721-E1A), empty control plasmid (SMMC-7721-vector), and cells without transfection (SMMC-7721) were combined with radiation ranging from 1-8 Gy. Cells were seeded into 96-well plates 24 h after irradiation. Following 48 h, as measured by the MTT assay, the viability of SMMC-7721-E1A cells had significant differences (P<0.05, Figure 3) with the two control groups when radiation dose was 2 Gy or more, indicating that the expression of E1A gene was sensitive to ionizing radiation. In addition, the dose-dependent effect of irradiation was noted. The viability differences between SMMC-7721-E1A cells and the other two control groups became greater as the radiation dose was increased. When radiation dose was under 8Gy, the viability of SMMC-7721-E1A cells decreased to only about 25% of control groups. 

*PEI-pcDNA3.1-E1A and cancer cells apoptosis*

**Figure 4:**
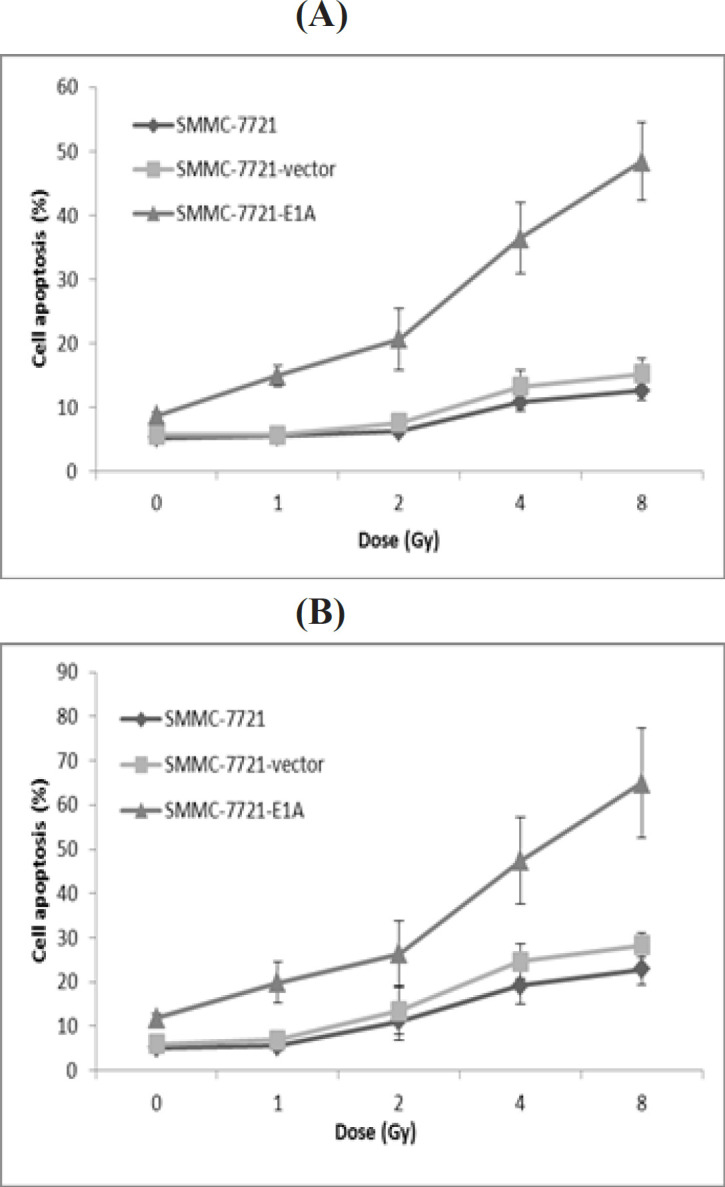
SMMC-7721, SMMC-7721-Vector and SMMC-7721-E1A Vells were Irradiated with Different Doses of Gamma Radiation Ranging from 1-8 Gy. After 24h or 72h incubation, cell apoptosis was measured by flow cytometry. (A) Cell apoptosis 24h after irradiation; (B) Cell apoptosis 72h after irradiation

To determine whether PEI-pcDNA3.1-E1A mediated decrease in viable cells occurred via apoptosis or not, SMMC-7721 cells were analyzed for apoptotic changes using flow cytometry. Under irradiation of 0 Gy, 1Gy, 2Gy, 4Gy, and 8Gy, the percentage of apoptotic cells in response to E1A-plasmid increased by about 3%, 9%, 9%, 25%, and 36% at 24 h and 7%, 14%, 15%, 28%, and 42% at 72 h, respectively, over SMMC-7721 control cells (Figure 4). The results were similar when compared with SMMC-7721-vector cells. 

*PEI-pcDNA3.1-E1A and cell cycle redistribution*

**Figure 5:**
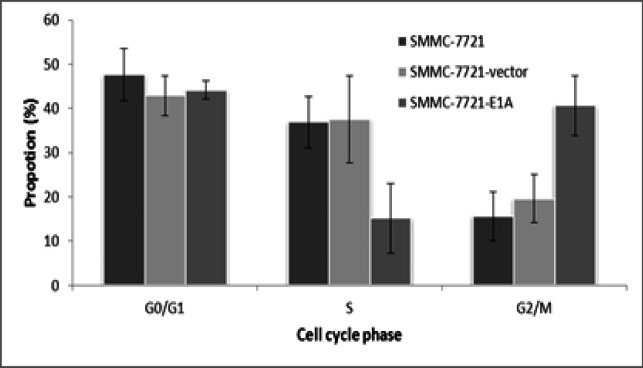
The Cell Cycle Distribution of SMMC-7721, SMMC-7721-Vector, and SMMC-7721-E1A Cells was Measured by Flow Cytometry

To study cell cycle distribution, SMMC-7721-E1A cells were analyzed for DNA content by using flow cytometry. Transfection with PEI-pcDNA3.1-E1A significantly increased the proportion of SMMC-7721-E1A cells in G2/M phase (P <0.01) and decreased the proportion of SMMC-7721-E1A cells in S phase (P<0.01), but no difference was found in G1 phase when compared with SMMC-7721-vector and SMMC-7721 cells (Figure 5).

## Discussion

The adenoviral vector is an ideal gene transfer system based on its capacity to produce high titers. As well as it being genomically stable, and having a low rate of integrating its own DNA into the host’s genome (Zhou et al., 2017). Although adenovirus is extensively used as the gene carrier in gene therapy, one of the main obstacles preventing the clinical application of Ad vectors is its interference with innate immune response. The most serious adverse event of adenovirus-based therapy is liver toxicity. Intravenously injected Ad vectors are predominantly sequestered by the liver, triggering hepatic inﬂammatory response that is characterized by acute transaminitis and vascular injury (Shayakhmetov et al., 2005). Given that proinflammatory cytokines IFN, IL-6, and TNF-α can be induced by adenoviral vectorsand *E1A* can sensitize cells to apoptotic killing by TNF-α, adenoviruses express wild-type *E1A* will have greater hepatotoxicity than E1-deleted adenovirus vectors (Engler et al., 2004). Accordingly, nonviral and noninfectious gene delivery system may be more suitable for treatment of hepatocellular carcinoma by using *E1A* gene . 

Non-viral vectors with cationic liposomes and polymers have been reported to date. Cationic vectors are known to exhibit high gene expression through specific mechanisms such as binding to the surface of cells, being taken up via the endocytotic pathway, and releasing of plasmid DNA (pDNA) from endosomes. However, it should be noted that cationic vectors cause undesirable gene expression and blood aggregation due to their cationic charges (Kodama et al., 2018). PEIs have been widely investigated as vehicles for nonviral gene delivery. They are positively charged polymers, which can form tight complexes with the negatively charged nucleic acid through spontaneous self-assembly. PEI-DNA complexes carry a net positive surface charge, which permits them to interact with the negatively charged cell membrane and internalize into cells (Godbey et al., 1999). Because of the high transfection efficiency in both in vitro and in vivo models, PEI is considered as the gold standard for polymer-based gene carriers (Godbey and Mikos, 2001). Unlike many viral vectors where repeated administration may be severely compromised by neutralizing antibodies, non-viral vectors, such as PEIs, are not associated with significant immune responses in the liver and may provide a more appropriate alternative for long term gene therapy. However, to date, there has been no study in English evaluating the ability and efficacy of PEI as the gene carrier of *E1A*. In our research, results on RT-PCR and Western Blot showed that we had successfully transfected E1A gene to SMMC-7721 cells through PEI-E1A plasmid. 

It is well established that *E1A* can sensitize cells to radiation (Lowe et al., 1993; Portella et al., 2003; Toth et al., 2003; Dilley et al., 2005; Idema et al., 2007), which was also confirmed by our results. When radiation dose was under 2Gy, there was a significant difference between *E1A* plasmid group and the other two control groups (Figure 3). It was also observed that the cell-killing effect was dose depended, indicating that *E1A* could remarkably increase the sensitivity of SMMC-7721cells to radiation. Moreover, the interaction of E1A gene systems and radiation was detected in a synergic manner. We also found that *E1A*-plasmid or irradiation alone could induce apoptosis, but combination of *E1A*-plasmid and irradiation was more effective than single treatment (Figure 4).

Multiple molecular mechanisms may contribute to the anti-tumor activity of *E1A* in different cancer types. It has been reported that E1A sensitizes cancer cells to apoptosis induced by ionizing radiation or chemotherapy through multiple pathways.

*E1A* gene expression has a signiﬁcant inhibitory effect on the cell proliferation of HeLa cells by inducing apoptosis through HER-2/Neu/Caspase-3 pathway (Shen et al., 2008). Most of the gene therapy research on E1A focuses on breast and ovarian tumor with positive *HER-2/Neu *expression. Given that SMMC-7721 cells are *HER-2/Neu* negative, there should be other pathways to explain the radiosensitizing effect of *E1A* gene.

Wild-type *p53* has a very short half-life which can be stabilized by *E1A* through up-regulation of p19ARF(de Stanchina et al., 1998), binding of CBP/p300 (Grossman et al., 1998), and proteasome modiﬁcation (Zhang et al., 2004). Thus, E1A will sensitize tumor cells to* p53*-dependent apoptosis in response to DNA damage (Debbas et al., 1993). The intratumoral delivery of exogenous *E1A* greatly increases *p53* expression and the radiosensitivity of cervical carcinoma in nude mice (Shen et al., 2010).

The inactivation of NF-κB may be one of the mechanisms of *E1A* mediated radio-sensitization (Shao et al., 1997). NF-κB can block the apoptosis induced by radiation. Activated NF-κB is one of the major pathways responsible for the radioresistance of melanoma cells (Munshi et al., 2004). *E1A* has been demonstrated to inhibit the activity of IκB kinase, thus prolonging the half-life of IκB, which is the major endogenous inhibitor of radiation-induced NF-κB (Shao et al., 2001).

Cell cycle regulation is an important factor to determine the radiation sensitivity of tumor cells (Yao et al., 2019). It is well known that different cell cycle phases have different radiosensitivity. Cells are most sensitive to irradiation during mitosis (M phase) and RNA synthesis (G2 phase), less sensitive in organelles synthesis (G1 phase), and least sensitive during DNA synthesis (S phase) (Pawlik et al., 2004). Our results showed that transfection of *E1A* synchronized SMMC-7721 cells to G2/M phase and decreased the proportion of cells in S phase. Therefore, it can be concluded that cell cycle redistribution may be another pathway of *E1A *to increase tumor’s sensitivity to radiation.

In summary, our study demonstrated that PEI-pcDNA3.1-E1A was capable of transfecting *E1A* gene to SMMC-7721 cells and enhancing the therapeutic effects of radiation. We also found that the* E1A*-mediated cell cycle redistribution may contribute to the E1A-mediated sensitization of radiation-induced apoptosis. These results presented support further development and research of PEI-pcDNA3.1-E1A in combination with radiotherapy for cancer therapy.
